# A fishing expedition to discover the pollinators of several subtropical Apocynaceae

**DOI:** 10.1002/aps3.11326

**Published:** 2020-02-13

**Authors:** Suzanne Koptur, Beyte Barrios, Imeña Valdes, Maha Nusrat

**Affiliations:** ^1^ Department of Biological Sciences and International Center for Tropical Botany Florida International University Miami Florida 33199 USA; ^2^ Pepin Academies Riverview Campus, 9304 Camden Field Parkway Riverview Florida 33578 USA; ^3^ Program in Plant Biology and Conservation Northwestern University 633 Clark Street Evanston Illinois 60208 USA; ^4^ Chicago Botanic Garden 1000 Lake Cook Road Glencoe Illinois 60022 USA; ^5^ The Institute for Regional Conservation 100 East Linton Boulevard, #302B Delray Beach Florida 33483 USA

**Keywords:** Apocynaceae, bees, butterflies, pollen, secondary pollen presentation

## Abstract

**Premise:**

Flowers of the Apocynaceae (milkweed family) have complex structures and pollination mechanisms. Pollen removal and deposition in *Angadenia*,* Pentalinon*, and *Echites* are similar, with anthers releasing pollen onto the sterile style head. The mid‐style head excretes a glue that coats the mouthparts of vistors to aid in the transfer of pollen. Subsequent probes may deposit pollen on the receptive stigmatic surface on the lowest part of the style head, with fertilization resulting after pollination by compatible pollen.

**Methods:**

By employing fishing line of different diameters, which reflected the diameters of the mouthparts of the different insect visitors, we determined the widths best able to remove and deposit pollen, thereby revealing which of the visitors could be effective pollinators, and which may be only nectar robbers.

**Results:**

We previously found that mouthpart (proboscis) width is correlated with pollen transfer effectiveness in *Angadenia berteroi* and confirmed this here in two other species, *Pentalinon luteum* and *Echites umbellatus*. Our data allowed the prediction of the most effective pollinators of these two other species.

**Discussion:**

The simulation of flower visitor mouthparts using fishing line can provide useful data for evaluating the potential for effective pollen removal and deposition by different visitors.

Flowers are a plant's way of saying “hi”—not to other plants, but to the animals that visit them for various reasons, inadvertently carrying their pollen to another plant of that same species in the course of their activities. Animals are attracted to flower color, odor, and shape, and many are rewarded for visiting by the provision of foods (edible floral parts, nectar, and pollen), fragrances, oils, resins, or places to shelter (Proctor et al., 1996; Willmer, 2011). To economize on the energy allocated to floral rewards, many plants have evolved flowers specialized to exclude some visitors, enabling them to save the rewards for those well suited to fulfill the role of a pollinator (Johnson and Steiner, 2000). Flowers with nectar rewards often have tubes containing secreted nectar at their bases, meaning only visitors with appropriately sized and shaped mouthparts can access the reward. For pollination, the visitor must also pick up pollen and deposit it on the receptive part of another flower, where, if the pollen is compatible, it can germinate and fertilize the ovules of the recipient flower (Richards, 1986).

In a review of studies of floral biology and pollination, more than half of all plant species considered were found to be visited by only one functional group, whereas in plant species with two or more functional groups of visitors, the groups often exerted similar selective pressures on floral morphology and traits (Fenster et al., 2004). From the plant point of view, the most important thing is whether the visitors pick up pollen and deposit it on other flowers (of the same species) (Armbruster, 2017). Some visitors are better than others at transporting pollen, as is the case with those visiting *Angadenia berteroi* (A. DC.) Miers (the pineland golden trumpet), which is visited by a variety of insects in four guilds (Barrios et al., 2016). Setting little fruit in the field, *A. berteroi* was found to be self‐incompatible in controlled hand‐pollination experiments (Barrios and Koptur, 2011), so not only were visitors needed for pollination, but pollen from unrelated plants was required for fruit set. In field studies of its pollination, there were 12 species of visitors to the flowers, but of those, only large, long‐tongued bees and gulf fritillary butterflies carried pollen (Barrios et al., 2016). These were less frequently seen to visit than were six species of skippers (Hesperiidae); those, however, were never observed with pollen on their bodies. Using a technique adapted from the hand‐pollination experiments conducted earlier (Darrault and Schlindwein, 2005; Barrios and Koptur, 2011), fishing line of diameters matching the width of the mouthparts of the various visitor guilds was used to compare the effectiveness of the different sizes of mouthparts in picking up pollen from flowers and depositing it on receptive stigmas (Barrios et al., 2016). The findings of that experiment corroborated single‐visit pollinator exclusion experiments, during which it was observed that only large bees carry enough pollen in a single visit to set a fruit.

A wide diversity of pollination syndromes and many kinds of floral visitors (mostly insects) have been documented in the Apocynaceae (Ollerton et al., 2019). Although many taxa with pollinaria have been well studied (e.g., *Asclepias* L., *Ceropegia* L., and *Cynanchum* L.), less is known about the reproductive biology of the non‐asclepioid members of the Apocynaceae family (Ollerton et al., 2019). The flowers of this diverse and species‐rich family exhibit a stepwise accumulation of floral characters, from the simplest structures in the Plumerioideae to the highly complex flowers of the Asclepiadoideae. In the majority of Apocynaceae species (>75%), including all in the APSA clade (Apocynoideae‐Periplocoideae‐Secamonoideae‐Asclepiadoideae; Endress et al., 2014), a gynostegium (structurally integrated style head and androecium) is present (Fishbein et al., 2018). In these flowers, the anthers are adnate to the corolla and form a conical structure surrounding the style head, and secondary pollen presentation occurs when the anthers transfer the pollen to the apical portion of the style head (Yeo, 1993).

In some subfamilies of the Apocynaceae, including those in which the study species occur (Echiteae [*Echites* P. Browne] and Odontadenieae [*Angadenia* Miers and *Pentalinon* Voigt]; Morales et al., 2017), the terminal portion of the style head is non‐receptive, functioning only to bear pollen in what can be called the pollen chamber; the middle area of the style head is a secretory area; and the receptive stigmatic area is at the base of the style head (Fig. 1). Pollen is deposited by the mouthparts of visitors searching for nectar in the flower. The floral visitor inserts its proboscis into the flower tube and exogenous pollen is captured at the receptive stigmatic area. When the proboscis is retracted, it slides past the mucilaginous part of the style head where the proboscis is coated with the mucilaginous substance, resulting in the pollen of that flower being picked up from the pollen chamber on the sterile style head to be carried away and deposited on a subsequent probe (Fig. 2). Studies have shown that this pollen‐gluing mechanism increases pollen transfer efficiency in other Apocynaceae that do not bear pollinaria (Livshultz et al., 2018).

**Figure 1:**
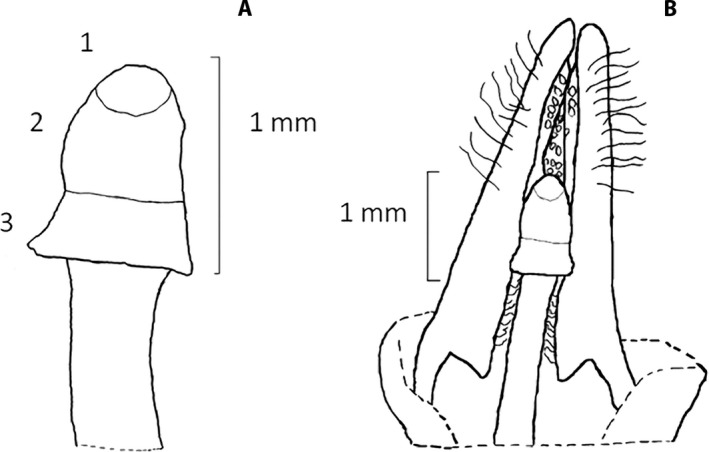
Style structure and location in *Angadenia berteroi*. (A) Diagram of the style head showing three zones: (1) the apical, sterile portion from which pollen is picked up; (2) the mid‐section where mucilage is produced and applied to mouthparts as they pass by, probing for nectar; and (3) the lower receptive stigmatic area, where pollen may be deposited as the mouthparts are retracted, prior to picking up pollen from this same flower. (B) The position of the style inside the anthers, forming the pollen chamber with its apical portion. Adapted from Barrios and Koptur (2011).

**Figure 2:**
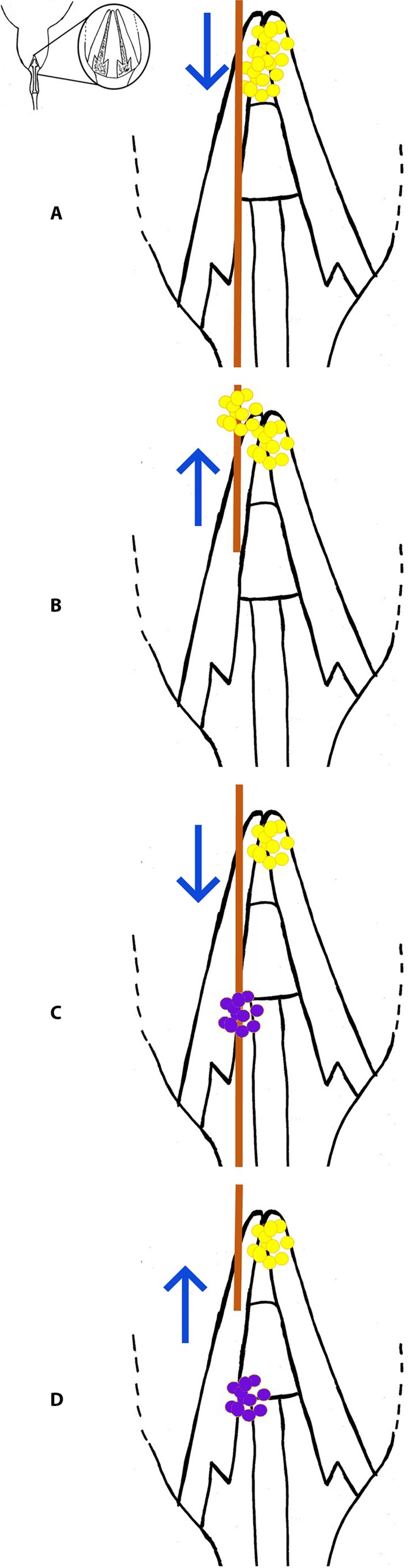
Schematic of an insect visitor proboscis collecting and depositing pollen in *Angadenia berteroi*. (A) Proboscis enters a flower to probe for nectar. (B) Proboscis picks up pollen from the style head as it is retracted from the flower. (C) Another probe from a proboscis bearing exogenous pollen. (D) Exogenous pollen is deposited on the receptive stigmatic surface as it is withdrawn. The position of the style in the flower is shown in the diagram in the upper left corner.

In this study, we used fishing line as a model proboscis to compare the effectiveness of different width proboscides in picking up and depositing pollen in the intact flowers of two members of the Apocynaceae (subfamily Apocynoideae, Echiteae, sensu Endress and Bruyns, 2000) native to the pine rocklands of southern Florida: *Pentalinon luteum* (L.) B. F. Hansen & Wunderlin and *Echites umbellatus* Jacq. Preliminary observations of the floral biology and flower visitors of these species were made in the Bahamas (Koptur et al., 2019), with further studies conducted in South Florida. The results of these experiments helped us predict which of the visitors we observed in the Bahamas and Florida could be effective pollinators of these two beautiful but little‐studied native plants.

## METHODS

### Study species

#### *Angadenia berteroi*

**Figure 3:**
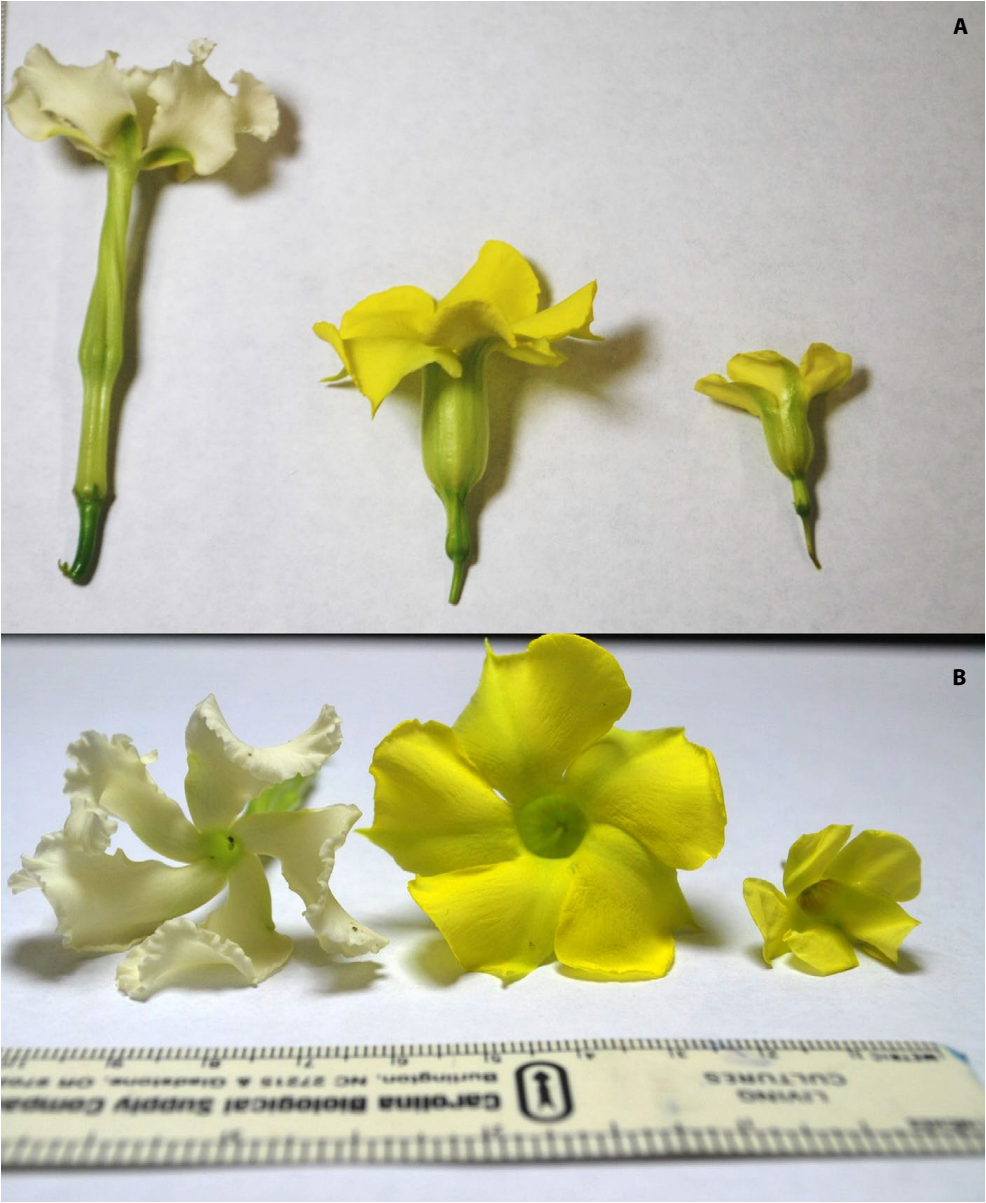
Flowers of the three study species. Longitudinal view (A) and face view (B) of *Echites umbellatus*,* Pentalinon luteum*, and *Angadenia berteroi* (from left to right).

Previous studies (Barrios and Koptur, 2011) demonstrated this species to be self‐incompatible, requiring pollinators for reproduction. The yellow flowers of *A. berteroi* (Fig. 3) are present from April to July, with a peak in April and May. The flowers open before sunrise and contain a concentrated nectar (30–67% sugar on a wt/wt basis). The fruits mature ca. 80 days after successful pollination, and the flower visitors are bees, butterflies, and skippers.

#### *Pentalinon luteum*

Observations in the Bahamas (Koptur et al., 2019) revealed this species to have flowers quite similar in appearance and structure to, but substantially larger than, those of *A. berteroi* (Fig. 3). The one pronounced difference is that *P*. *luteum* anthers have long appendages that are twirled together when the flowers open and protrude from the bell of the corolla, somewhat like the lolling tongue of a dog. These plants are also apparently self‐incompatible (Koptur, unpublished data). The flowering peak of this species is spring and summer, although flowers can be found on plants throughout the year. The flowers open at sunrise and have a nectar concentration slightly lower than, but in the same range as, *A. berteroi* (ranging from 25% to 41% sugar, with a mean of 34.2%; Koptur et al., 2019). The fruits take 100 days to mature after successful pollination.

#### *Echites umbellatus*

Markedly different in appearance from *Angadenia* and *Pentalinon*,* E. umbellatus* flowers are white with a very long, spiraling floral tube (Fig. 3). Observations in the Bahamas revealed a flowering peak in summer, although flowering individuals may be observed throughout the year. No visitors were ever observed there, although hawkmoths were expected/predicted due to the color, morphology, and nocturnal and diurnal opening time of the flowers (Koptur et al., 2019). A single flower may last up to 10 days, and each contains a large nectar reward with concentrations similar to that of *Pentalinon* (20–31% sugar, with a mean of 26%; Koptur et al., 2019). Most *E. umbellatus* individuals are self‐incompatible, but some individuals are self‐compatible (Koptur, unpublished data). The fruits mature at least 100 days after successful hand pollination.

Vouchers of the studied plant species were deposited at Fairchild Tropical Botanic Garden (FTG) and the Academy of Natural Sciences at Drexel University (PH). Vouchers of the insect visitors are retained in the first author's collection at Florida International University (FIU) and will be deposited in the Arthropod Collection at the Florida Museum of Natural History.

### Pollinator effectiveness prediction

To determine how the thickness of the mouthparts of each visitor type affects pollen transfer effectiveness, the length and width of each captured visitor's proboscis were measured using a dissecting microscope (Leica MZ12 5; Leica Microsystems, Wetzlar, Germany). Pinned insect specimens were also observed from the museum collection at the Gerace Research Centre, a research station of the University of the Bahamas on San Salvador Island, and their mouthparts were measured. These measurements were made in fall 2014 and spring 2015, and the Bahamas specimen measurements were taken in June 2015.

The experiments were conducted using fresh flowers harvested from plants grown from seeds in the greenhouse at FIU. These seeds were collected from plants in natural areas of South Florida. The flowers were picked and carried in egg cartons to the lab table, where the experiments described below were carried out in uniform conditions. The experiments began in January 2014 and were completed in June 2015.

Four weights and diameters of nylon monofilament fishing line (4 lb, 0.20‐mm diameter; 6 lb, 0.23‐mm diameter; 8 lb, 0.28‐mm diameter; 25 lb, 0.53‐mm diameter) were cut into 12‐cm lengths and inserted into single flowers to simulate flower probes by insects. These different diameters were chosen to correspond to the average width of the mouthparts of the four different groups observed by Barrios et al. (2016) to visit *A. berteroi* flowers (from narrowest to widest: skippers, non‐skipper butterflies, short‐tongued bees, and long‐tongued bees). These same diameters were used here because they represent a range of mouthpart sizes, including hawkmoth proboscides, the widths of which may be within the range of the three narrowest fishing line widths used.

**Figure 4:**
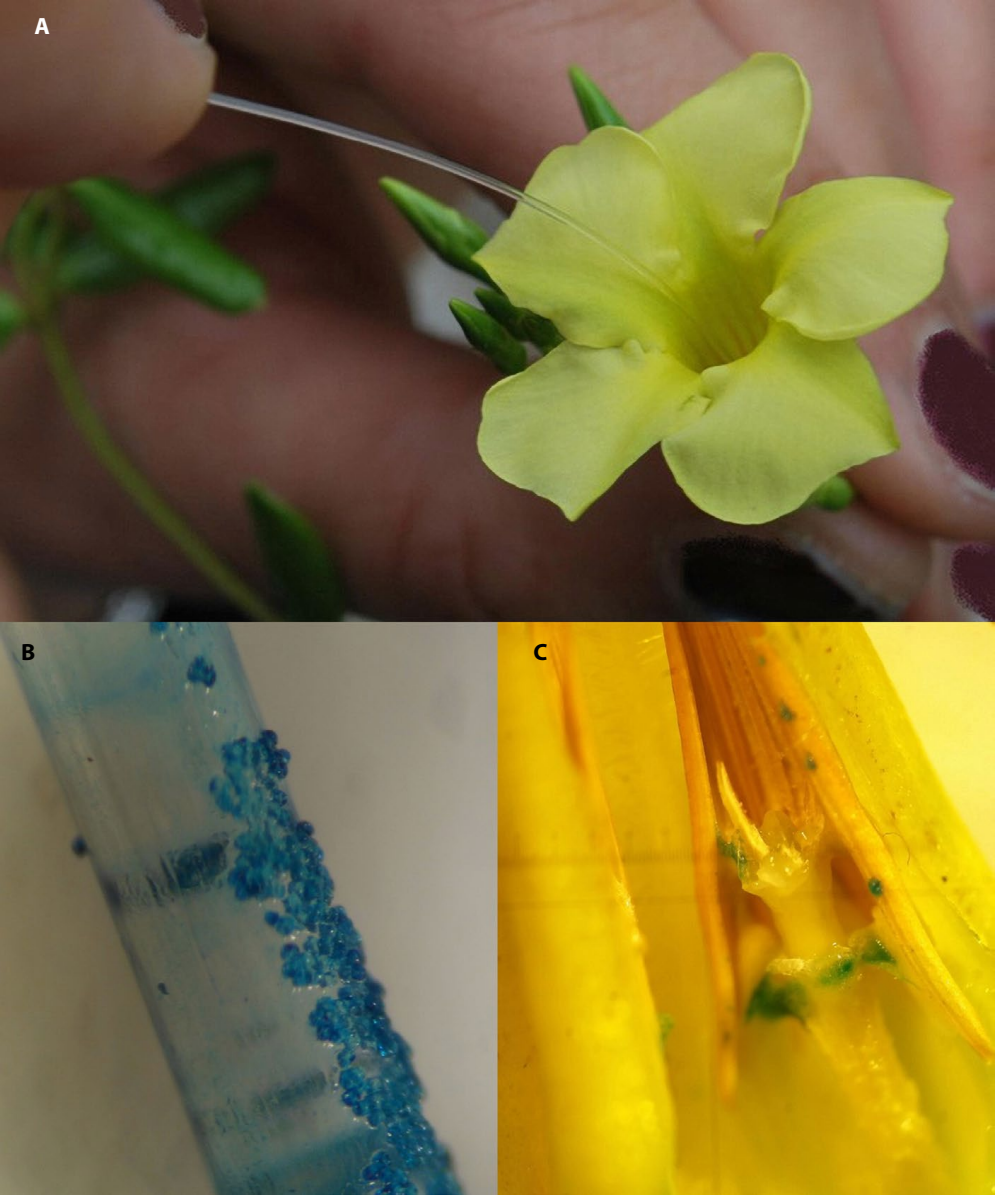
Experimental simulation of pollen removal and pollen deposition. (A) Fishing line introduced into flower by hand. (B) Pollen deposited on fishing line after one probe (insertion and withdrawal), stained with methylene blue. (C) Dissected flower corolla showing stigmatic area stained after the insertion of a fishing line with stained pollen grains.

For each probe, a single piece of new, unused fishing line was inserted into a fresh flower (from greenhouse‐grown plants from multiple populations) until it reached the bottom of the corolla tube (where it might contact the sticky secretions of the middle portion of the style head), then carefully withdrawn to avoid dislodging any adhering pollen grains (Fig. 4A). The number of pollen grains adhering to each line were then counted under the dissecting microscope to determine whether the thickness of fishing line corresponded to the number of pollen grains removed. A total of 50 replicates were performed for each line diameter for each plant species.

To determine whether fishing‐line thickness was related to pollen deposition, individual flowers of each species were hand pollinated using new segments of unused fishing line in the same four different diameters used above. A 12‐cm length of fishing line was inserted to the bottom of the corolla tube of a fresh flower to collect pollen (as described above). The fishing line and the adhering pollen grains were stained with a 1.5% solution of methylene blue (Fig. 4B), applying a drop to the pollen‐bearing area of the line, then blotting the extra liquid onto a paper towel, and then inserted into another fresh flower. The flowers that received the stained fishing line were then dissected, and the length (in millimeters) of the stigmatic surface area that was stained blue was measured (Fig. 4C). That length indicated the proportion of the stigmatic surface encountered by the stained pollen adherent to the fishing line, a proxy for pollen deposition on the stigma. At least 20 replicates were performed for each line diameter on each plant species, using a fresh flower every time (23 each for *Angadenia*, 20 each for *Pentalinon*, and 22 each for *Echites*, for a total of 92, 80, and 88 flowers for each species, respectively).

An ANOVA was used to test for differences among the proboscis lengths and widths of different visitor groups, as well as between the lengths of the stigmatic surface stained with methylene blue. Post hoc tests were conducted using Tukey's honestly significant difference (HSD) test to identify differences between pairs of visitor groups. Pollen loads on the fishing line were compared using the Kruskal–Wallis test and a post‐hoc Mann–Whitney test to determine differences in the pollen loads among the fishing line sizes, as the data were not normally distributed. A sequential Bonferroni correction was used to control for type I errors in all pairwise comparisons. Statistical analyses were performed using SPSS 21 (SPSS, 2014).

## RESULTS

As previously observed in the pine rocklands of South Florida (Barrios et al., 2016), the long‐tongued bees on San Salvador Island had the thickest mouthparts of all tested insects (Table 1). The widest mouthparts, measured in millimeters, were observed in *Xylocopa cubaecola* Lucas (mean ± SD: 0.82 ± 0.22 mm), followed by *Centris versicolor* Fabricius (0.66 ± 0.02 mm). The mouthparts of the short‐tongued bees were not much narrower (*Megachile bahamensis* Mitchell: 0.59 ± 0.08 mm, *M. alleni* Mitchell: 0.64 ± 0.09 mm, and *Agapostemon columbi* Elliott: 0.67 ± 0.12 mm). The widest fishing line used was 0.53 mm in diameter, which is close to, but slightly narrower than, all of these measurements. Because of this, the fishing line widths used in this experiment do not clearly distinguish between the long‐ and short‐tongued bee visitors.

**Table 1:** Mouthpart width measurements of specimens in the insect collection at Gerace Research Centre.

Order/Family	Species	*n*	Mouthpart width (range, mm)	Mean (mm)	SD
Hymenoptera					
Anthophoridae	*Centris versicolor* Fabricius	5	0.64–0.69	0.66	0.02
Anthophoridae	*Xylocopa cubaecola* Lucas	15	0.54–1.37	0.82	0.22
Halictidae	*Agapostemon columbi* Elliott	13	0.54–0.88	0.67	0.12
Megachilidae	*Megachile alleni* Mitchell	10	0.51–0.79	0.64	0.09
Megachilidae	*Megachile bahamensis* Mitchell	5	0.50–0.71	0.59	0.08
Lepidoptera					
Heliconiidae	*Agraulis vanillae insularis* Maynard	2	0.49–0.50	0.50	0.01
Heliconiidae	*Dryas iulia* Fabricius	3	0.28–0.36	0.33	0.04
Hesperiidae	*Epargyreus zestos zestos* Geyer	4	0.39–0.51	0.43	0.06
Hesperiidae	*Polygonus leo* Savignyi	2	0.28–0.29	0.29	0.01
Hesperiidae	*Urbanus proteus domingo* Scudder	4	0.24–0.28	0.28	0.02
Nymphalidae	*Anartia jatrophae* Johansson	3	0.19–0.24	0.21	0.03
Papilionidae	*Battus polydamus lucayus* Rothschild & Jordan	4	0.38–0.47	0.41	0.04
Papilionidae	*Heraclides andraemon bonhotei* Sharpe	2	0.28–0.38	0.33	0.07
Pieridae	*Ascia monuste eubotea* Latreille	5	0.26–0.34	0.29	0.03
Pieridae	*Phoebis agarithe antillia* Brown	6	0.24–0.46	0.31	0.09
Pieridae	*Phoebis sennae* L.	6	0.27–0.40	0.33	0.06

Note

*n = n*umber of individuals sampled.

The Lepidoptera mouthparts were narrower than those of the Hymenoptera visitors, with the widest being the Antillean gulf fritillary (*Agraulis vanillae insularis* Maynard, 0.50 ± 0.01 mm). The mean mouthpart widths of the other butterflies ranged from 0.19 to 0.43 mm, with the three skippers (Hesperiidae) in the middle of the range.

**Figure 5:**
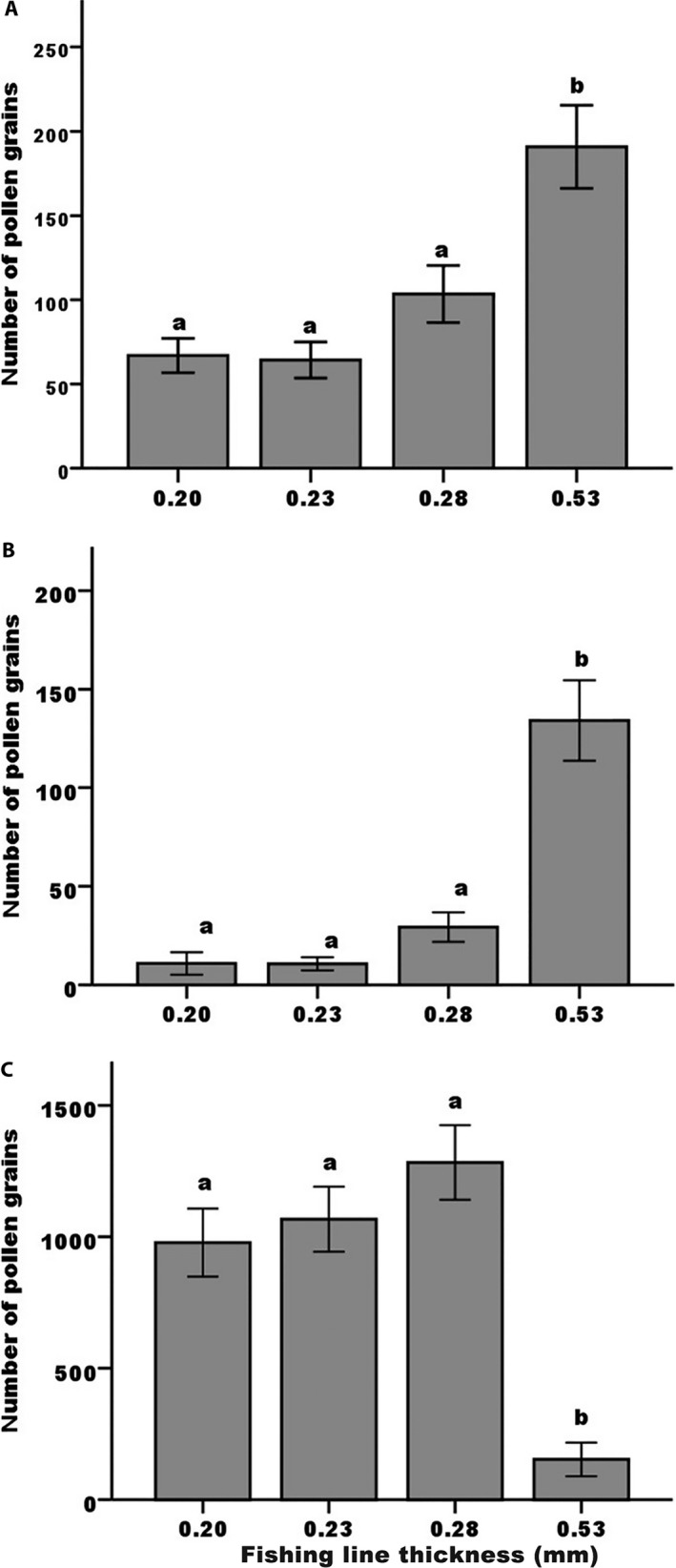
Pollen picked up on fishing lines of different widths inserted into flowers of (A) *Angadenia berteroi*, (B) *Pentalinon luteum*, and (C) *Echites umbellatus*. Fishing line widths corresponded to the four groups of visitors, as described by Barrios et al. (2016): 0.20 mm, skippers; 0.23 mm, non‐skipper butterflies; 0.28 mm, short‐tongued bees; 0.53 mm, long‐tongued bees (although the diameters of the mouthparts of the two bee groups in the present study overlapped). Hawkmoth proboscis widths range from approximately 0.2–0.4 mm. Different letters above the bars indicate significant differences (Kruskal–Wallis test; *n *=* *50 for each group). Plotted are medians and standard errors.

Fishing lines of different diameters were used to replicate the size of the mouthparts of the four visitor groups. In *A. berteroi*, the line with the widest diameter picked up significantly more (roughly twice as many) pollen grains than the three smaller widths (*X* ^2^_3,79_ = 20.7, *P *<* *0.0001, *n *=* *172; Fig. 5A). The same pattern was even more substantially seen in *P. luteum* (*X* ^2^_3,79_ = 19.63, *P *<* *0.0001), with the widest‐diameter line picking up four times more pollen grains than the narrower ones (Fig. 5B). We found precisely the opposite pattern in *E. umbellatus* (*X* ^2^_3,79_ = 22.99, *P *<* *0.0001), where the thinner‐diameter lines were all much more effective at removing pollen than the widest‐diameter line (Fig. 5C).

**Figure 6:**
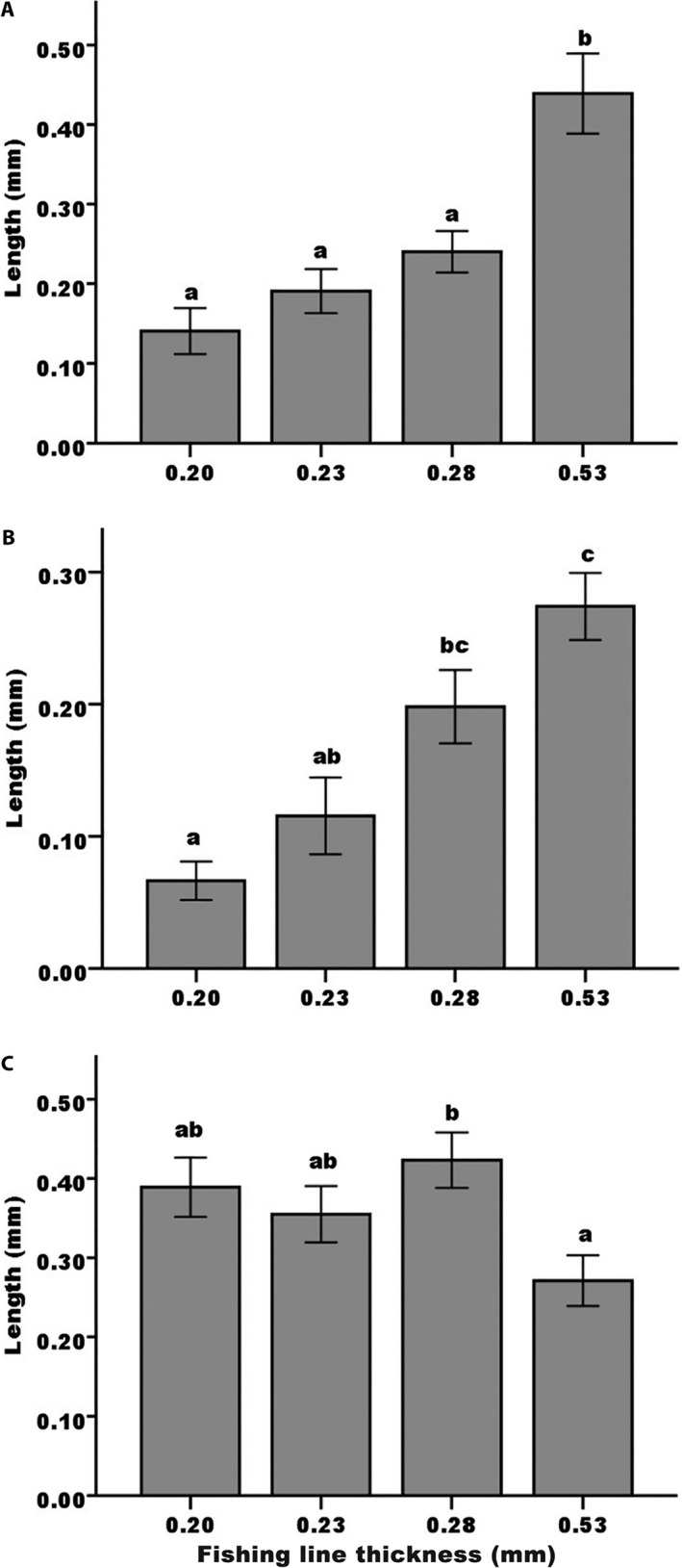
Length of the stigmatic surface stained by a probing with fishing line covered with stained pollen grains. Plant species and fishing line widths were as in Fig. 5: (A) *Angadenia berteroi*,* F*_3,51_ = 14.19, *P *<* *0.0001; (B) *Pentalinon luteum*,* F*_3,79_ = 13.57, *P *<* *0.0001; and (C) *Echites umbellatus*,* F*_3,87_ = 3.46, *P *=* *0.02. Different letters above the bars indicate significant differences (Tukey post‐hoc comparisons; *N *=* *23, 20, and 22). Plotted are means and standard errors.

As for implications for pollen deposition, the widest fishing line stained the widest swath of the stigmatic surface of *A. berteroi* (when compared with the narrower ones, *F*_3,51_ = 14.19, *P *<* *0.0001; Fig. 6A). The pattern was not as clear in *P. luteum* (*F*_3,79_ = 13.57, *P *<* *0.0001), with each adjacent width not significantly different from the next, although the widest width stained substantially more of the stigmatic surface than the narrower two (Fig. 6B). The results for *E. umbellatus* were not significant (Fig. 6C), with several of the widths (the two narrowest and the widest) not significantly different from each other (*F*_3,87_ = 3.46, *P *=* *0.02). In this species, the mid‐width line stained the widest swath, suggesting a greater pollen deposition.

## DISCUSSION

The importance of proboscis length in effective pollination by bees has been demonstrated (Inouye, 1980; Dohzono et al., 2004; Arbulo et al., 2011), whereas floral tube length determines which species can transfer pollen in hawkmoth‐pollinated plants (Alexandersson and Johnson, 2002; Moré et al., 2007; Anderson et al., 2010). Corolla width in the flowers of various species has been shown to correspond to pollination effectiveness (Galen, 1989; Campbell et al., 1996; Moré et al., 2007). The importance of mouthpart diameter has not received the same attention, however, perhaps because in many pollination systems the pollen is transported on other parts of the visitor's body. In plants with flowers that have pollen borne within the floral tube, the thickness of the proboscis is as important as its length. From the perspective of the animal visitor, the length of its proboscis determines whether it can reach the nectar reward or whether it is excluded (or must rob the flowers to get the nectar). In flowers where the mouthparts must contact the anthers and stigma within the floral tube, the width of the mouthparts may be even more important.

The surface of an insect's proboscis is different from the surface of nylon fishing line, and we acknowledge this distinction. However, as both surfaces can receive the glue from the mid‐region of the style head of the flowers, we considered fishing line a reasonable simulation of a proboscis. The pollen held in the pollen chamber on the terminal sterile part of the style head adheres to that glue and can be deposited on the receptive part of a style head on a subsequent probe. By using the fishing line as a “glue receiver,” we approximated the phenomenon of probing by an insect's proboscis.

There was a significant relationship between fishing line width and pollen removal from these flowers; in *Angadenia* and *Pentalinon*, the widest line was most effective, whereas in *Echites*, the thinner widths picked up more pollen. This suggests that visitors with specific mouthpart widths are more effective at picking up pollen when probing flowers for nectar. The results of the pollen deposition/stigma staining were not quite as clear, but at least in the first two species, the wider lines stained substantially greater areas of the stigma, indicating that visitors with wider mouthparts would likely deposit more pollen in a single visit.

The importance of a pollinator to a plant is influenced by both its ability to disperse pollen grains to conspecific stigmas and its visitation frequency (Waser et al., 1996; Reynolds et al., 2009; Ne'eman et al., 2010). Our results helped us to determine that large bee visitors to *A. berteroi* flowers were the most effective pollinators, and also indicate that other visitors with narrower mouthparts might also pollinate effectively with more visits per flower. Although no skippers were found to have pollen on their mouthparts when collected in the field, the width of their proboscis was adequate to collect some pollen in our experiment. Perhaps repeated probes by the same insect, or multiple visits by different individuals, could deposit enough pollen for adequate pollination and fruit set.

The information we have from these experiments suggests that large bees are likely to be the most effective pollinators of *P. luteum*. The shape of the corolla is very similar to that of *A. berteroi*, with a wide bell and a narrow tube at the base, allowing access to the nectar with a relatively short proboscis (short compared with that of a butterfly or hawkmoth). On San Salvador Island in the Bahamas, we observed many large butterflies visiting *Pentalinon* flowers, in addition to bees. Several butterfly species were found to carry *Pentalinon* pollen on their mouthparts (Koptur et al., 2019), including the Antillean gulf fritillary. Of all the Lepidoptera measured, the proboscis of the Antillean gulf fritillary is the widest and closest in size to the wider mouthparts of long‐tongued bees. Four other butterflies were observed visiting *Pentalinon* flowers in the Bahamas: *Ascia monuste* L., *Dryas iulia* Fabricius, *Phoebis agarithe* Boisduval, and *P. sennae* L. (Koptur et al., 2019). Some were found to carry *Pentalinon* pollen, however, the narrower width of the mouthparts of many butterflies and skippers implies they would not be as effective at the removal and deposition of pollen as large long‐tongued bees. We observed the long‐tongued bee *X. cubaecola* visit *Pentalinon* flowers in the Bahamas, and although we were not able to capture specimens to examine their mouthparts for pollen, we measured their dimensions from previously collected specimens. The width of their mouthparts corresponds to the widest fishing line, which we showed here removes and deposits significantly more pollen than other sizes, suggesting that this large long‐tongued bee is an important pollinator for *P. luteum* in the Bahamas. In the lower Florida Keys, an introduced long‐tongued orchid bee was observed to be a common visitor to *P. luteum* (B. Harris, Florida International University, personal communication).

Although we spent many hours observing *E. umbellatus* in the Bahamas (Koptur et al., 2019), in South Florida pine rockland fragments, and in the Everglades National Park (Koptur, unpublished data), we have not yet seen any visitors to the flowers. The long, spiral corolla tubes and the effectiveness of the thinner diameter fishing line in removing pollen suggests that hawkmoths are its likely pollinators, as they possess long tongues narrow enough to reach the nectar at the base of the long floral tubes. Considering that individual flowers remain open for seven days or more and that fruit is produced in unmanipulated plants in the field, we can assume that we may have not been looking at the right flowers at the right times; after all, one quick visit from a pollen‐carrying moth could bring enough pollen for fruit set. We have one record of a deceased hawkmoth (*Agrius quinquemaculata* Haworth) that was discovered by W. Villavicencio (Florida International University) hanging from its tongue, stuck in a flower that had fallen from the plant, draped over the vining plant stem—a sad sight, but evidence that this species of hawkmoth visits *E. umbellatus* in South Florida and carries pollen on its proboscis.

We believe that others might find this fishing line technique useful, not only for simulating proboscides in hand‐pollination experiments, but in comparing the abilities of different groups of visitors in the pick up and deposition of pollen from various plant species. When combined with field observations of visitor behavior and frequency of visits, as well as experimentation allowing visits to pristine flowers and comparing pollen deposition and fruit set, the results of the fishing line pollen removal and deposition experiments can help determine the most effective pollinators of a particular plant species.
